# Mimicry in viceroy butterflies is dependent on abundance of the model queen butterfly

**DOI:** 10.1038/s42003-019-0303-z

**Published:** 2019-02-18

**Authors:** Kathleen L. Prudic, Barbara N. Timmermann, Daniel R. Papaj, David B. Ritland, Jeffrey C. Oliver

**Affiliations:** 1 School of Natural Resources and the Environment, University of Arizona, Tucson, AZ 85721 USA; 2 Department of Entomology, University of Arizona, Tucson, AZ 85721 USA; 3 Department of Medicinal Chemistry, University of Kansas, Lawrence, KS 66045 USA; 4 Department of Ecology & Evolutionary Biology, University of Arizona, Tucson, AZ 85721 USA; 5 Department of Biology, Erskine College, Due West, SC 29639 USA; 6 Office of Digital Innovation & Stewardship, University Libraries, University of Arizona, Tucson, AZ 85721 USA

## Abstract

Mimics should not exist without their models, yet often they do. In the system involving queen and viceroy butterflies, the viceroy is both mimic and co-model depending on the local abundance of the model, the queen. Here, we integrate population surveys, chemical analyses, and predator behavior assays to demonstrate how mimics may persist in locations with low-model abundance. As the queen becomes less locally abundant, the viceroy becomes more chemically defended and unpalatable to predators. However, the observed changes in viceroy chemical defense and palatability are not attributable to differing host plant chemical defense profiles. Our results suggest that mimetic viceroy populations are maintained at localities of low-model abundance through an increase in their toxicity. Sharing the burden of predator education in some places but not others may also lower the fitness cost of warning signals thereby supporting the origin and maintenance of aposematism.

## Abstract (web summary)

Kathleen Prudic et al. examine the persistence of mimicry in viceroy butterflies in locations with low model abundance. They show that when queen butterflies are less abundant, viceroy butterflies become more abundant, but also increase their chemical defenses to gain protection from predation.

## Introduction

Mimetic relationships, some of the most dramatic examples of natural selection, are the consequence of interactions between model, mimic, and selective agent(s). Relative frequencies of models and mimics in both space and time play a profound role in how a mimicry relationship evolves and persists^1–3^. Variation in local abundance can lead to different selection regimes, especially when there are significant differences in profitability among the species involved in the mimetic relationship^4–6^. This variation among selective regimes promotes local adaptation, resulting in potentially different evolutionary trajectories among populations within a species^7,8^. There are several predicted outcomes when a more profitable mimetic species experiences a reduction in model abundance: mimics may remain, but also at low abundance, especially when predation is weak^9,10^; mimics could go extinct due to intense predation invited by their conspicuousness^11^; or alternative defensive strategies such as crypsis or disruptive coloration could evolve in the palatable mimetic species^5,12^.

The well-studied mimicry between queen (*Danaus gilippus* Cramer) and viceroy (*Limenitis archippu*s Cramer) butterflies in Florida^13–15^ is ideal for addressing how model abundance influences mimic abundance and defense. Viceroys have been described both as Batesian mimics^13^ and Müllerian co-models of queens^14,15^ based on predator palatability experiments. Both viceroys and queens exhibit spatial variation in abundance and palatability across Florida^15–17^; however, little is known about how queen abundance may influence viceroy abundance, chemical defenses, and palatability^18^. Both species acquire chemical defenses from their respective larval host plants, although queens and viceroys feed on plants in distant families (Apocynaceae and Salicaceae, respectively). This difference in larval hosts results in different chemical defense profiles in adults: queens sequester steroidal, or cardiac, glycosides from their larval hosts, milkweeds and milkvines^15^, whereas viceroys sequester phenolic glycosides and their derivatives by consuming their larval hosts, willows, and poplars^18^. Both cardiac^19^ and phenolic glycosides^20^ are known to be unpalatable, noxious, and sometimes toxic to natural enemies such as herbivores, parasitoids, and predators.

Here, we integrate the results of population surveys, chemical analyses, and predator behavior assays to assess the influence of queen abundance on the dynamics of this mimicry system. We sampled queens and viceroys from eight sites across peninsular Florida to explore how mimic abundance and chemical defense are influenced by model abundance at a local scale. We demonstrate viceroys occur in high abundance in northern Florida, where queen abundance is low, correlating with the rarity of the queen’s primary larval host plant, a result contrary to predictions of mimicry theory. Exploring explanations for this contrary result, we also demonstrate a striking spatial relationship between viceroy chemical defense and queen abundance: viceroys have greater chemical defense and lower palatability in areas of low-queen abundance. Variation in viceroy chemical defense and palatability is independent of variation the chemical defense of the larval host plant. These observations support a previously unexplored consequence of a decrease in model abundance—mimics themselves may become more unprofitable to predators in regions where mimics shoulder most of the cost of predator education.

## Results

### Model and mimic abundance varies across peninsular Florida

**Fig. 1:**
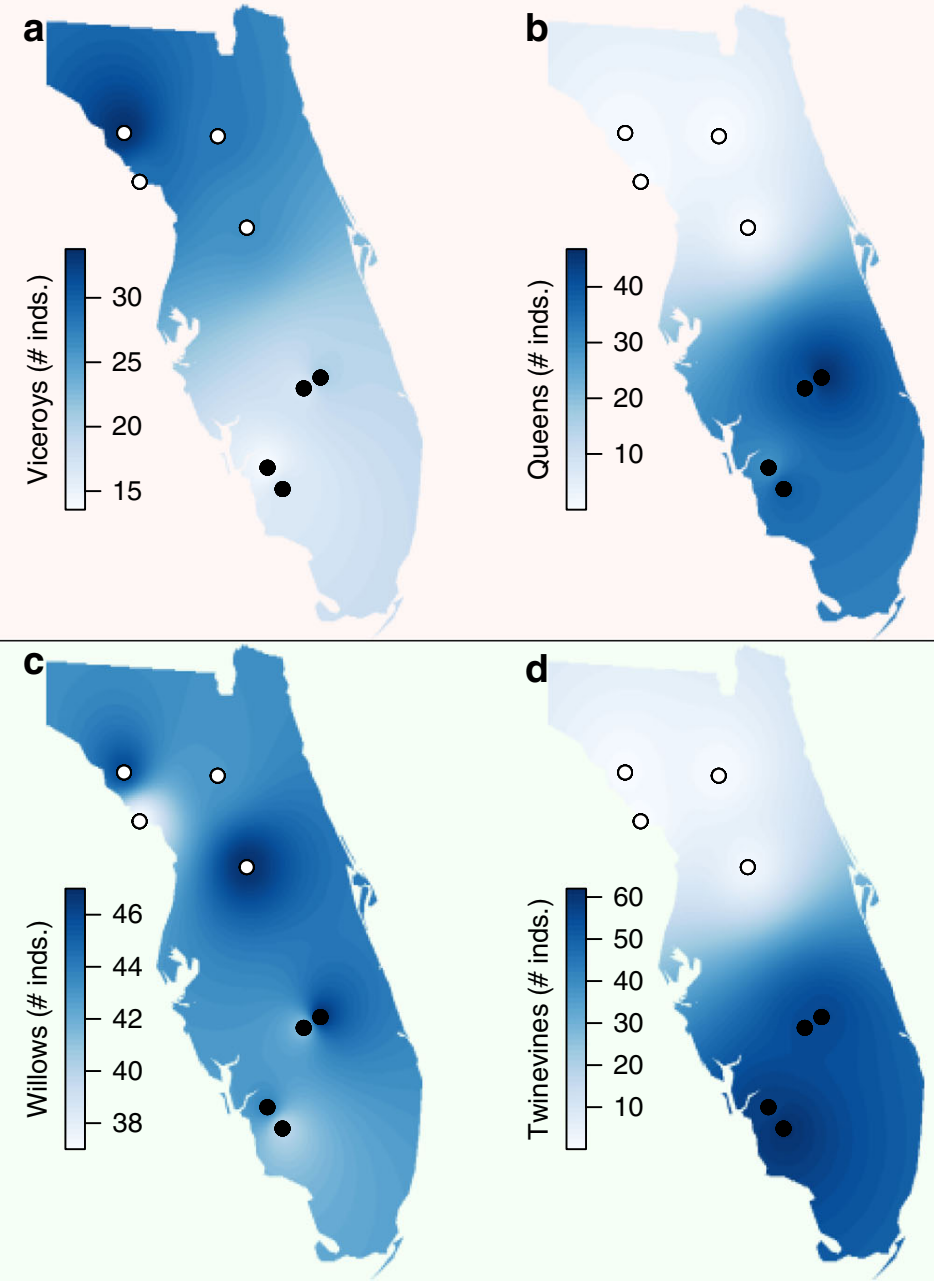
Florida viceroy butterflies occur without their queen butterfly models. **a** Viceroy abundance is greater in northern Florida, while **b** queen abundance is high in southern Florida with occasional individuals found in northern Florida, **c** Carolina willow (*Salix caroliniana*), a prominent viceroy larval host plant, is found in high abundance across Florida; **d** white twinevine (*Funastrum clausum*), a prominent queen larval host plant, is found in high abundance in southern Florida, but is nearly absent from the northern half of Florida. In this and subsequent figures, open circles indicate those sites with low-queen abundance and filled circles indicate those sites with high queen abundance. Shading reflects extrapolation based on inverse distance weighting

Queens and viceroys are broadly distributed across much of the Florida peninsula, but the relative abundances of each across this region were previously described in general terms^18^. Given the importance of model abundance in understanding mimicry dynamics, we surveyed sites across the Florida peninsula to more precisely quantify the abundances of the queen, the viceroy, and each insect’s respective larval host plants. Viceroys were found across all surveyed sites, with slightly lower abundance in southern Florida (northern sites: 28.00 ± 0.87 [mean ± SE] adult viceroys per hour; southern sites: 16.44 ± 0.67 adult viceroys per hour); in contrast, queens occurred at much lower abundance in the northern sites (Fig. 1a, b) (northern sites: 0.13 ± 0.06 adult queens per hour; southern sites: 37.13 ± 1.22 adult queens per hour). Contradicting conventional predictions of mimicry theory, viceroy abundance was negatively correlated with queen abundance (*N* = 72, *F* = 11.412, *p* = 0.002, generalized linear mixed-model) (Fig. 1a, b). That is, the abundance of the mimic was higher in regions of low-model abundance, the opposite of the predicted response if the mimic bears more of the burden of predator education^21^. An alternative model for the viceroy, the monarch, does occur across Florida at least seasonally, but monarchs were never observed at any of the eight study locations or sampling periods (*N* = 72). The viceroy’s primary host plant, the Carolina willow (*Salix caroliniana* Michaux), exhibited no pattern of spatial variation in abundance (Fig. 1c) while the queen’s primary host plant in southern Florida^22^, white twinevine (*Funastrum clausum* (Jacquin) Schultes), was rare in the northern portion of our sampling area (Fig. 1d). The rarity of white twinevine likely accounts for the low abundance of breeding queens in northern Florida (*N* = 72, *F* = 4.794, *p* = 0.041, generalized linear mixed-model) (Fig. 1b, d).

### Viceroy chemical defense increases with low model abundance

**Fig. 2:**
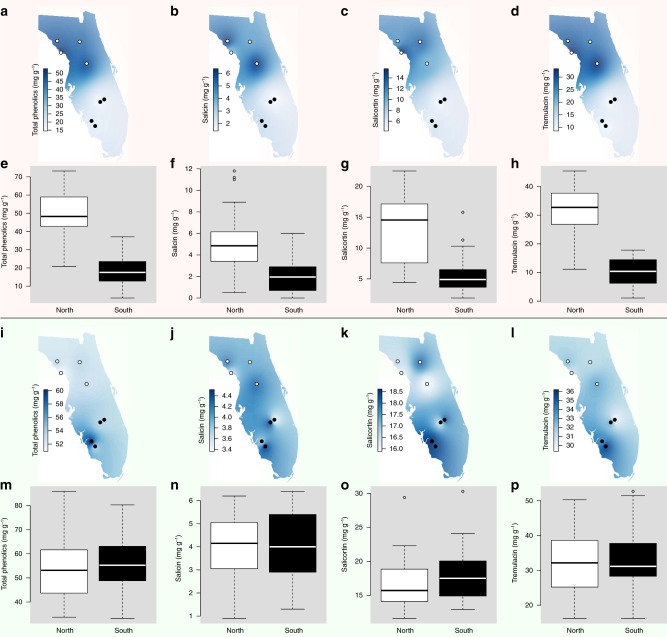
Viceroy populations with fewer queens have greater levels of constitutive phenolic chemical defensive compounds. Viceroys from northern Florida populations, where queen abundance is low, contain higher levels of nonvolatile phenolic compounds than do southern Florida viceroy populations (**a**, **e** total phenolics; **b**, **f** salicin; **c**, **g** salicortin; **d**, **h** tremulacin) (*N* = 64 samples), while levels of these same compounds remain consistent in Carolina willow populations across Florida (**i**, **m** total phenolics; **j**, **n** salicin; **k**, **o** salicortin; **l**, **p** tremulacin) (*N* = 64 samples). For box plots, horizontal lines in boxes indicate the first, second, and third quartiles and whiskers show the extreme upper and lower observed values within 1.5 times the interquartile range (IQR). Circles in boxplots show observed values beyond 1.5× IQR

**Fig. 3:**
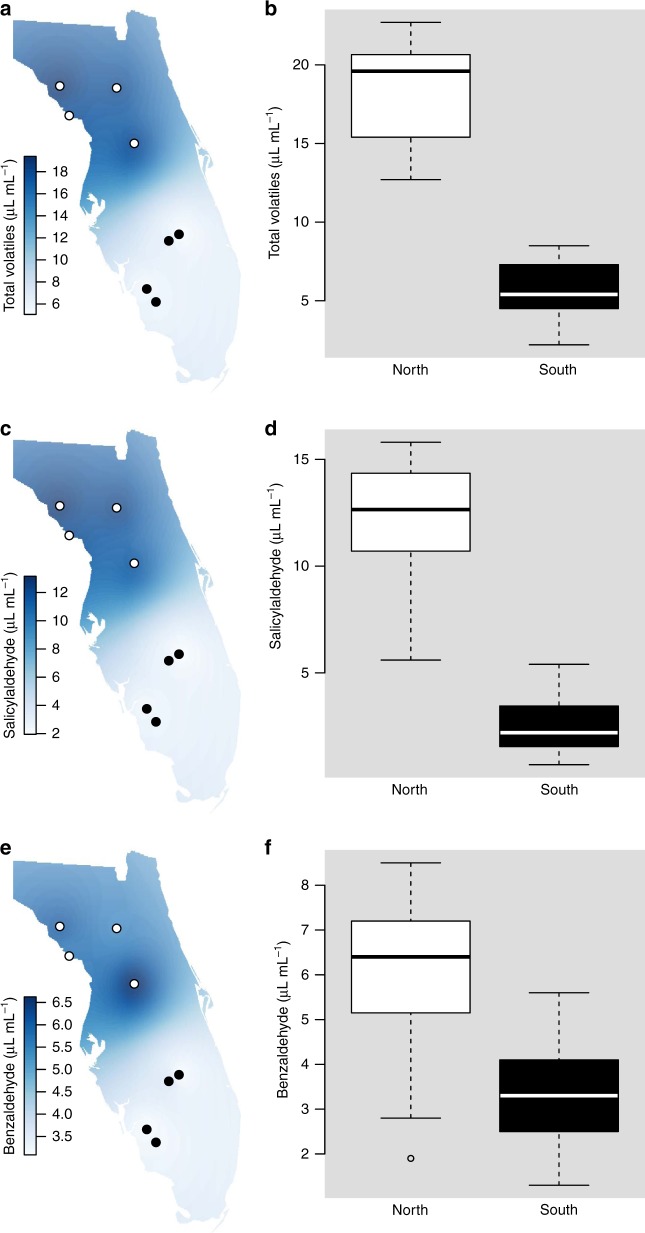
Viceroy populations with fewer queens have greater levels of volatile phenolic chemical defensive compounds. Viceroys sampled from northern Florida populations contain higher levels of volatile phenolic compounds than do southern Florida viceroy populations (**a**, **b** total volatile phenolics; **c**, **d** salicylaldehyde; **e**, **f** benzaldehyde) (*N* = 64 samples). For box plots, horizontal lines in boxes indicate the first, second, and third quartiles and whiskers show the extreme upper and lower observed values within 1.5 times the interquartile range (IQR). Circles in boxplots show observed values beyond 1.5× IQR

In northern sites where queens were absent or in low abundance, the high abundance of viceroys requires an explanation: how does the mimic thrive in regions without its model? We investigated the potential for increased chemical defenses of the viceroy as a possible mechanism of persistence in regions of low-model abundance, and tested possible reasons for the observed variation in viceroy chemical defense. Variation in host plant chemistry may be driving viceroy chemical defense and palatability, as observed in other willow-feeding insects^23,24^. However, while viceroy chemical defense varied across Florida in relation to queen abundance, Carolina willow chemical defense was consistent across the sampled sites (Fig. 2). Viceroys in locations with low-queen abundance (few to no models) had greater nonvolatile phenolic chemical defenses than those in locations with high-queen abundance (Fig. 2a–h; Table 1). Viceroys also release bitter chemical volatiles when disturbed^18^; these compounds, too, were all greater in viceroys from locations with low-queen abundance (Fig. 3; Table 1). A similar pattern is observed when, instead of queen abundance, the abundance of the queen’s larval host plant, white twinevine, is used to predict viceroy chemical defense: for all seven measures of viceroy chemical defense, viceroys had higher defenses in areas with low abundance of white twinevine (Table 2). While butterfly distributions are often attributed to the distribution of their respective larval host plant species, the pattern described here shows the model host plant distribution influences the mimic species, similar to patterns found in other mimicry systems^5^. Changes in viceroy chemical defenses were not related to variation in Carolina willow total phenolics (*F*_1,40.94_ = 0.163, *p* = 0.6883), salicin (*F*_1,57.96_ = 0.385, *p* = 0.5372), salicortin (*F*_1,57.25_ = 0.286, *p* = 0.5949), or tremulacin (*F*_1,55.47_ = 0.447, *p* = 0.5065) (Fig. 2i–p) in linear mixed-model tests. Thus, viceroy chemical defense was higher in the regions of low-model abundance, but this increase in butterfly defensive compounds was independent of variation in the viceroy larval host plant.

**Table 1:** Queen abundance predicts viceroy chemical defense

	Compound	Parameter estimate	df	*F*	*p*
Nonvolatiles	Total nonvolatile phenolics	−0.8	5.584	95.276	0.0001
	Salicin	−0.083	6.373	23.564	0.0024
	Salicortin	−0.191	8.369	17.608	0.0027
	Tremulacin	−0.519	6.251	72.64	0.0001
Volatiles	Total volatile phenolics	−0.3	5.843	81.144	0.0001
	Salicylaldehyde	−0.234	5.731	81.751	0.0001
	Benzaldehyde	−0.07	6.29	48.074	0.0001

Values are results of linear mixed-models testing the effect of queen abundance on viceroy phenolic chemical defenses

**Table 2:** Queen larval host plant abundance predicts viceroy chemical defense

	Compound	Parameter estimate	df	*F*	*p*
Nonvolatiles	Total nonvolatile phenolics	−0.552	61	142.611	<0.0001
	Salicin	−0.055	6.02	19.45	0.0045
	Salicortin	−0.134	6.011	30.696	0.0015
	Tremulacin	−0.363	62	134.926	<0.0001
Volatiles	Total volatile phenolics	−0.221	6.009	380.08	<0.0001
	Salicylaldehyde	−0.173	6.011	341.74	<0.0001
	Benzaldehyde	−0.049	62	64.776	<0.0001

Values are results of linear mixed-models testing the effect of white twinevine abundance on viceroy phenolic chemical defenses

### Viceroy unpalatability increases with low-model abundance

**Fig. 4:**
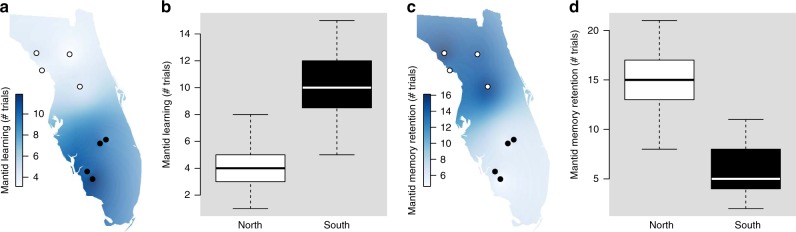
Viceroy populations with fewer queens are easier to learn to avoid and harder to forget for predators. **a**, **b** Mantid predators learn to avoid viceroys from northern Florida faster than they learn to avoid viceroys from southern Florida (*N* = 64). **c**, **d** Mantid predators’ memory retention is shorter when experienced with viceroys from southern Florida than when experienced with viceroys from northern Florida (*N* = 64). For box plots, horizontal lines in boxes indicate the first, second, and third quartiles and whiskers show the extreme upper and lower observed values within 1.5 times the interquartile range

We investigated if this observed increase in viceroy chemical defense was correlated with greater protection from natural enemies. Learning and memory retention are hallmark behavioral responses of predators to unpalatable prey^21,25,26^. As unpalatability increases, the rate of predator aversion learning should also increase. In other words, the predator should require fewer experiences to learn to avoid more noxious prey^21,25^. Similarly, predator memory retention should increase as palatability decreases^25,26^. To assess viceroy palatability, we measured the rate of aversion learning and the length of memory retention using Chinese mantids (*Tenodera sinensis* Saussure) as a model predator known to respond to viceroy chemical defenses^18^. Both attributes were influenced by the population of origin of the viceroys. Mantids given viceroys from populations with low-queen abundance learned to avoid them faster (*N* = 64, *F*_1,6.7_ = 47.457, *p* = 0.0003, linear mixed-model) (Fig. 4a, b) (northern sites: 4.00 ± 0.32 trials until avoidance; southern sites: 10.44 ± 0.45 trials until avoidance) and retained their avoidance behavior longer than did mantids given viceroys from populations with high-queen abundance (*N* = 64, *F*_1,6.34_ = 75.593, *p* < 0.0001, linear mixed-model) (Fig. 4c, d) (northern sites: 15.03 ± 0.60 days until reattack; southern sites: 5.88 ± 0.44 days until reattack). Thus, the observed pattern in viceroy palatability follows the same pattern of viceroy chemical defense, with northern sites characterized by low-queen abundance and chemically defended, unpalatable viceroys and southern sites characterized by high-queen abundance and relatively palatable viceroys with lower chemical defense.

## Discussion

We have shown high abundance of viceroy mimics in locations with low-queen model abundance (Fig. 1), increased levels of the mimic’s defensive compounds in locations with low-model abundance (Figs. 2 and 3), and decreased mimic palatability in locations with low-model abundance (Fig. 4). Most importantly, we found a correlation between decreased queen abundance and increased viceroy chemical defense and unpalatability. On the face of it, these findings appear inconsistent with predictions of conventional mimicry theory reviewed in refs. ^4,27^, and contribute to growing empirical evidence suggesting that mimetic species occur, persist, and even thrive in areas of low-model abundance^5,12,28,29^. In addition to reconciling assignment of this system as both Batesian^13^ and Müllerian^14^, the work presented here has several implications for our understanding of the evolution and dynamics of mimicry and warning coloration.

Our findings help to explain how physiological and ecological circumstances could account for a mimic’s transition from Batesian to Müllerian. The viceroy presumably evolved from a palatable, nonwarningly colored ancestral form to one resembling aposematic *Danaus* species and was thus originally a Batesian mimic^5,30^. This interpretation is derived from both experimental work demonstrating North American relatives of the viceroy are relatively palatable^31,32^ and the near ubiquity of cryptic or disruptive coloration, as opposed to warning coloration, throughout the *Limenitis* clade, in which the viceroy is deeply nested^5,30^. When viceroys and queens are both relatively abundant in the same Florida location, the viceroy is a Batesian mimic as originally described^13^ being quite palatable and minimally chemically defended (filled circles in Figs. 2–4). Queen butterflies are known to vary in both chemical defense and palatability, with the most chemically defended being centered in Broward County of southern Florida^15^. However, in locations with low-queen abundance, viceroys are unpalatable and unprofitable to predators (open circles in Figs. 2–4) and have been described as co-models, or Müllerian mimics, with the queens^14,18^. The geographic variation in relative abundances of model and mimic described here presents an explanation of how viceroys transitioned from palatable Batesian mimics to unpalatable Müllerian co-models: lacking the protection of unprofitable queens in the northern part of their range, viceroys may have evolved their own means of chemical defenses.

The variation in defensive strategies employed by viceroys in this study also illustrates how spatial variation in mimicry relationships could ultimately result in the transition from a dishonest mimic to an honest aposematic signaler reviewed in refs. ^3,4^. The pathway to aposematism is often framed as evolution from a cryptic unprofitable form to a conspicuous unprofitable form driven by selective advantages of advertising unprofitability. However, this transition is problematic. Predators must learn to associate novel warning signals with unprofitability, usually at some survival cost to the bearer of the signal. Until learning happens, bearers of the warning signal are especially vulnerable by virtue of being conspicuous. If the number of signalers in the population is sufficiently small, the process of predator education may lead to elimination of the warning signal from the population. The paradox of a conspicuous signal arising and persisting in the face of a significant burden of predator education can be resolved if individuals bearing such signals share this burden with a co-occurring model species with a similar signal. In this way, the initial fitness cost of an emergent warning signal in a mimetic species may be reduced in the presence of a model species, allowing the signal to persist and spread through a population.

These results, especially the relationship between queen abundance and viceroy chemical defense, highlight several important drivers influencing the dynamics of mimicry systems. First, the observed variation in chemical defense and profitability of both models and mimics can only be understood in the context of how the variation is geographically distributed. Theoretical models of mimicry systems should accommodate spatio-temporal variation in defensive strategies, and additional empirical work would be useful in understanding how ecological and evolutionary factors contribute to this variation. For example, measuring the strength of selection, rates of gene flow between Florida viceroy populations, and potential for plasticity in viceroy chemical defense profiles would inform the mechanism underlying the reported pattern, as would investigations into the relative benefits and potential costs of allelochemical sequestration, chemical defense, and unpalatability in both viceroys and queens^33–35^. Furthermore, given potential variation in sensory capabilities among different members of the predator community, investigations evaluating responses of predators, especially vertebrates, would also be useful in understanding the factors influencing the mimicry continuum^36,37^.

Another important conclusion from this work is how relative abundances of models and mimics at a local scale influence defensive strategies. While discussions of mimicry often involve categorical statements of the presence or absence of model species^12,27^, such a perspective would fail to explain the observed distribution of chemical defenses in viceroys. In the viceroy-queen mimicry system, both species occur throughout the southeastern United States and would thus be considered sympatric. Without a quantitative approach to measure abundance, the presence of queens throughout Florida would preclude understanding the factors driving variation in viceroy chemical defense and predator learning^38^. Finally, the distribution of the queen’s primary host plant, white twinevine, as a predictor of viceroy chemical defense illustrates the impact that third-party interactions may have on mimicry systems. Such interactions may involve bottom-up limitations of resources required by model species^5^ as well as top-down pressures exerted by predator communities^39^. This deeper characterization of mimicry environments offers an opportunity to expand future mimicry research to include spatial and temporal ecological interactions for a better understanding of how and when mimicry evolves and persists.

## Methods

### Geographic locations and survey techniques

We surveyed and collected specimens three times (June, July, and September) for 2 years (2003 and 2004) at eight locations in Florida, USA^18^. From south to north, the locations were Corkscrew, Collier County (26.361, −81.519); Lehigh Acres, Lee County (26.560, −81.678); Lake Istokpoga, Highlands County (27.296, −81.296); Cornwell, Highlands Country (27.396, −81.120); Leesburg, Lake County (28.788, −81.895); Cedar Key, Levy County (29.214, −83.021); Gainesville, Alachua County (29.637, −82.200); and Jena, Dixie County (29.667, −83.185) (Fig. 1). Relative abundance of each species was measured by calculating the rate of capture per species per hour per person. Two field surveyors sampled each site and the same two individuals surveyed all the sites. One surveyor sampled queens and the other sampled viceroys, switching their target species after 1 h. Twenty individuals were kept for chemical defense and palatability studies; any additional individuals were released once the survey was complete. Each field site was sampled for 2 h continuously along a 400 m × 10 m transect on sunny days between 900 and 1600 h. The number of larval hosts was also recorded at each site along the transect: the Carolina willow (*Salix caroliniana*) for the viceroy and the white twinevine (*Funastrum clausum*) for the queen butterfly. In separate analyses, we tested for an effect of queen abundance on viceroy abundance, an effect of *F. clausum* on queen abundance, and an effect of *F. clausum* on viceroy abundance (see Statistical Analyses, below).

### Chemical analyses of viceroys and host plants

We investigated geographic variation in the defensive phenolic glycosides in the viceroy butterfly and its larval host plant, the Carolina willow^18^. Four samples of butterflies and four samples of willow were collected from each of the eight population locations in July 2003 and July 2004. A butterfly sample consisted of 5 adults (either all male or all female) (~0.5 g dry weight), while a willow sample consisted of 16 young leaves, two leaves from eight different plants (~10 g dry weight). All specimens were weighed, then air-dried at room temperature for 1 week and reweighed. Whole butterflies including their wings were used in the extraction. The extraction and identification protocol is described in through detail in previous research^18^. Analyses of each sample were conducted using an Agilent 1100 HPLC system tandem with Agilent MSD-Trap-SL ion trap mass spectrometer with the samples identity blinded from the technician. Calibration curves were constructed for the three phenolic compounds using the liquid chromatography–mass spectrometry (MS) protocol described above. For each compound, a characteristic product ion was chosen from its MS/MS as its quantification ion. Peak integration and quantification were performed automatically using Agilent Chemstation software (version A.10.01). The same samples were run twice for quantification to ensure consistency within a sample. The concentrations were considered consistent if runs 1 and 2 were within 10% of each other. If not, then the sample was re-injected until the two runs reached the consistency criteria. However, only the concentration of the first run was used for reporting and statistical analyses (*N* = 64 willow samples; *N* = 64 viceroy samples). In separate analyses, we tested for an effect of queen abundance on defensive phenolic glycosides in viceroys and an effect of queen abundance on defensive phenolic glycosides in willows. Each of these tests involved four different analyses that differed only in their response variable: total phenolic glycosides, salicin, salicortin, and tremulacin. We also tested for an effect of willow defensive phenolic glycosides on viceroy defensive phenolic glycosides. Note that we restricted these tests between willow and viceroy phenolic glycosides to a single compound or category. That is, we tested for an effect of total phenolic glycosides in willow on total phenolic glycosides in viceroys, an effect of willow salicin on viceroy salicin, an effect of willow salicortin on viceroy salicortin, and an effect of willow tremulacin on viceroy tremulacin. In testing the relationship between willow and viceroy defensive chemistry, we used the mean value of the concentration for each site/date combination as the predictor of the concentration of the corresponding compound in viceroys. For example, the total phenolic concentration in the two willows sampled at Corkscrew on 1 July 2013 was 62.8 and 38.1 mg g^−1^. We used the average, 50.45 mg g^−1^, as the predictor for the total phenolic concentration observed in viceroys.

We evaluated whether or not the chemical profile of the viceroy defensive secretion varied by geographic location. Butterflies were caught at the eight field sites in July 2003 and July 2004, stored live at 8 °C, and analyzed within the next 3 days before being fed. The defensive secretion was sampled directly from the abdomen of the butterfly using a glass capillary^18^. Three males and three females were sampled from each site, and each individual butterfly secretion was analyzed separately (*N* = 64). Sample identity was blinded from the technician. Volatile compounds were quantified by the external standard method using a six-point standard curve with standards ranging from 0.005 to 5.0 mL mL^−1^. Calibration curves from triplicate injections of 2.0 ml were obtained using the gas chromatography (GC)–MS protocol above. Peak integration and quantification were performed automatically using Saturn 2100 Workstation software. Two millilitre of the secretion were collected from a disturbed butterfly and dissolved in 2.0 mL of ethyl acetate with 1.0 mL of 0.25 M p-chlorotoluene as the internal standard. Then 2.0 mL of this solution were injected directly into the GC column. Each butterfly sample was run on the GC twice for consistency. The concentrations were considered consistent if runs 1 and 2 were within 5% of each other. If not, then the individual butterfly was resampled until the two runs reached the consistency criterion. However, only the concentration of the first of those two runs was used for reporting and statistical analyses (*N* = 64). We tested for an effect of queen abundance on volatile defensive phenolics in viceroys; this involved a total of three separate analyses that differed only in the response variable: total phenolic volatiles, salicylaldehyde, and benzaldehyde.

### Predator behavioral experiments

Laboratory-reared adult Chinese praying mantids, *Tenodera sinensis* (Mantodea: Mantidae) served as the experimental predator^18,26^. Chinese mantids are a known predator of butterflies and perform well in laboratory experiments. They are naturalized in the U.S. and have been observed preying on viceroys and queens at study locations in Florida and respond behaviorally to unpalatable prey similar to avian predators^14,26^. Fifteen egg cases were purchased from Carolina Biological Supply Company and reared to adults in 2003 and 2004. Mantids were reared in individual cages on a diet of fruit flies, houseflies, true bugs, and crickets. Mantids did not have access to butterfly species or distasteful prey before the experimental feeding trials. Each mantid was fed two adult crickets every night throughout the aversion learning and memory retention experiments.

All behavioral experiments were conducted in a laboratory arena consisting of three components: a rectangular perch for the predator, a square floor, and a cylindrical wall. The entire arena was painted a dark uniform gray^26^. The arena was illuminated by three full-spectrum halogen lamps (Solux-Eiko, 50 W, 4700 ^o^K, 36^o^ field of illumination). Each lamp was positioned 23 cm above the highest point of the perch and 20 cm from the other lamps. In all experiments, a trial began by placing a single mantid at the top of the perch inside the arena wall, such that the mantid’s longitudinal axis was perpendicular to the long axis of the perch. The mantid was allowed to acclimate for 5 min before trials began. All mantids remained at the top of the perch for all experiments and trials. Viceroys collected in July 2003 and July 2004 were used in this experiment and their identity was blinded from the human observer. Abdomens, rather than the entire insect, were used for consistency with previous experiments involving viceroys and avian predators^14,28^ and to remove potentially confounding effects of wing pattern and size variation^40,41^. Viceroy abdomens are black with white stripes, while the other butterfly abdomens used (*Pieris rapae* and *Vanessa cardui*) in the experiment were uniformly either white or light brown. A single butterfly abdomen was introduced to the arena by attaching one end of a string to a dowel then slowly dropping the attached abdomen from above within the field of view of the mantid. In separate analyses, we tested the effect of queen abundance on each of the two measures of palatability: predator learning aversion rate and predator memory retention.

The predator learning assay compared rates at which mantids learned an aversion for viceroys originating from different sites^18,26^. A single originally naïve mantid (*N* = 64) was fed only viceroys from a single locality. A trial ended either 5 min after a mantid attacked and ate the abdomen, or, if the mantid did not attack the abdomen, 5 min after the abdomen was presented to the mantid. After the trial ended, the butterfly abdomen or its remains were removed. If the mantid attacked the abdomen, it was returned to its holding cage after 2.5 min. If the mantid did not attack the abdomen, it was presented with a known palatable butterfly abdomen of similar size (*Vanessa cardui* or *Pieris rapae*) after 2.5 min to evaluate its hunger status. If the mantid attacked the palatable abdomen, it was evaluated as hungry. The mantid was not allowed to feed on the palatable abdomen because the abdomen might serve as a positive reward negatively affecting the aversion learning trials. This protocol prevented the mantid from associating its response to the viceroy abdomen with a palatable reward. A mantid was considered to show an aversion to the viceroy abdomen when it oriented to but failed to attack the abdomen, and subsequently attacked a palatable abdomen, in three consecutive trials. To test for a geographic difference between prey palatability and predator aversion learning rate, the number of trials until mantids reached aversion criterion was compared between geographic locations. All mantids were fed two crickets every evening in their cages to control hunger levels across treatments.

We evaluated predator memory retention by measuring the number of days until the mantid re-attacked a viceroy abdomen after reaching aversion criterion^18,26^. We compared predator memory retention among sites to evaluate the relative palatability of viceroy butterflies to predators. The same mantids (*N* = 64) used in the learning experiment were tested 2 days after the day they met the aversion criteria above and retested every second day thereafter. A trial ended either when the mantid attacked and consumed a viceroy, or when the mantid oriented to a viceroy, failed to attack, but subsequently attacked a palatable butterfly abdomen. A mantid was considered to have lost its aversive response when it attacked and partially or completely consumed a viceroy abdomen. All mantids were fed two crickets every evening in their cages to control hunger levels across treatments.

### Statistical analyses

All analyses were performed in the R software package^42^, with the lme4^43^ and lmerTest^44^ packages. For all analyses, we used generalized linear mixed-effect models, including collection year and site as random intercept effects. In analyses where the response variable was continuous, we used the lmer function of lmerTest and used the Satterthwaite method to approximate degrees of freedom. In analyses where the response variable was ordinal (i.e., abundance), we used the glmer function of the lme4 for Poisson regression. Degrees of freedom are not reported for the Poisson regression models, as they are not available for the glmer function^43^. For purposes of display and reporting means, we classified sites using *K*-means clustering based on the number of adult queens observed in abundance assays. Applying the elbow method to determine the number of clusters based on the total within sum of squares, *K*-means clustering identified two clusters: one cluster consisted of the four northern sites, with low queen abundance, and the other cluster consisted of the four southern sites, with high-queen abundance. Maps were created in R with inverse distance weighting using the sp^45,46^, gstat^47^, rgdal^48^, and raster^49^ packages.

### Code availability

R scripts for all analyses can be found at https://github.com/jcoliver/viceroy-mimicry-geography and are archived on Zenodo^50^.

### Reporting summary

Further information on experimental design is available in the Nature Research Reporting Summary linked to this article.

## Supplementary information

Reporting Summary

## Data Availability

Data for all analyses can be found at https://github.com/jcoliver/viceroy-mimicry-geography and are archived on Zenodo at 10.5281/zenodo.1469783^49^. Figures 1–4 have associated raw data and the data are unrestricted for use^49^.
